# Fabrication and Characterization of Acicular Micro-Textured Copper Sheet Device for Low-Temperature Heat Radiation

**DOI:** 10.3390/mi14030507

**Published:** 2023-02-22

**Authors:** Tatsuhiko Aizawa, Hiroki Nakata, Takeshi Nasu

**Affiliations:** 1 Surface Engineering Design Laboratory, Shibaura Institute of Technology, Tokyo 144-0045, Japan; 2 Ebina Denka Kogyo, Co., Ltd., Tokyo 144-0033, Japan

**Keywords:** low-temperature heat radiation, acicular micro-texturing, high absorbance from near-IR to far-IR wavelengths, micro-cone unit shape, Mie resonance

## Abstract

An acicular microtextured sheet was developed as a heat radiation device from the high-temperature source to the cooling medium in the infrared (IR) spectrum. The copper surface was modified by acicular micro-texturing to place a semi-regular micro-/nano-cone structure onto it. FT-IR (Fourier transformation IR) spectroscopy was utilized to measure the transmittance diagram in near-IR to far-IR wavelengths. The wavelength (λ) of 6.7 μm, where the highest absorbance valley was detected in the diagram, was equivalent to the doubled size of the micro-cone average height, with H_ave_ = 3.3 μm; λ ~ 2 × H_ave_. The electromagnetic waves in the far-IR wavelength were emitted by acicular micro-textured metallic sheets. The heat radiation transfer experiment was performed to describe this low-temperature heat radiation behavior. No temperature rise was detected on the black-colored polycarbonate (BC-PC) plate away from the bare copper sheet without textures, located on the high-temperature source. The temperature increased by 4 K on the BC-PC plate using the acicular textured copper sheet device. The emitter temperature also decreased significantly by 50 K or 50% of the heat source temperature.

## 1. Introduction

A fuel cell-engine radiator suffers from the difficulty of the far lower heat transfer than is demanded in engineering requirements [1]. The huge heat waste generated in LIDAR (light detection and ranging laser imaging detection and ranging) turns to be another engineering issue of nuisance in heat transfer [2]. Heat radiation in a low temperature provides a way to solve those challenging problems [3]. The heat radiation mechanism has been used as an efficient heat transfer system from the high-temperature source in nature and in technology [4], e.g., the solar system for electric power generation [5], electric heating units [6], and the daytime radiation cooling system [7]. This electromagnetic wave emission process was theoretically and experimentally analyzed in the literature [8], e.g., Plank’s law for frequency distribution in the spectrum, Wien’s displacement law for temperature dependency, and Stephan–Boltzmann’s law for the effect of a temperature rise to the emitted power. After these laws, high-energy heat radiation requires a higher temperature source. Hence, new innovative ideas are necessary to put low-temperature heat radiation into practice.

Nano-photonic control [9,10] and meta-material design [11,12] have provided a solution to thermal radiation transportation with wavelength selectivity. Those methods stood on the usage of selectively patterned nano-structures of photo-voltaic materials. The regularly aligned micro-cavity-textured surface enabled heat radiation with the equivalent wavelength to the micro-cavity depth [13,14]. The temperature of the micro-cavity-textured emitter was reduced by 10% of the heat source temperature in various experiments [14]. Those studies imply that micro- and nano-textured aluminum and copper sheets work as a resonator to emit electromagnetic waves with selective wavelengths from the heat source. The first idea of this low-temperature thermal emission was invented as a Mie resonance device in [15] and an IR antenna in [16]. Those device and antenna designs stood on the metallic microstructure with a size comparable to the IR wavelengths in some micro meters. Each microstructure played a role as a micro-bolometer to detect IR waves with a fast response. The array of the micro-pillars on the IR lens and IR window was also expected to be working as an anti-reflecting layer, as suggested in [17].

**Figure 1:**
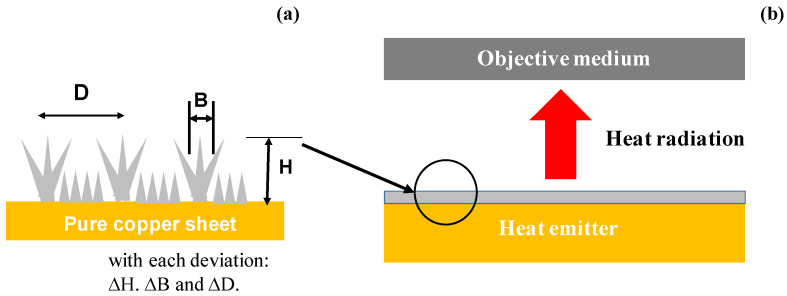
A schematic view on the emission of electromagnetic waves in the near-IR to far-IR wavelengths through the micro-cone-shaped metallic surface. (**a**) Acicular microtextures designed for arrayed IR antenna onto the emitter and (**b**) heat radiation system from the emitter to the objective medium through the acicular microtextures.

A new heat radiation device is proposed in the present study to emit electromagnetic waves in near-IR to far-IR wavelengths from the acicular microtextured surface layer with the dimension of micro-cone unit cells, ranging from sub-μm to 10 μm in size, as depicted in Figure 1a [18,19]. Through this microtextured layer, the objective medium is heated by a radiation mechanism from the heat emitter, as illustrated in Figure 1b. Several microtextured copper sheet specimens are prepared to have a different unit size and alignment by using electro-chemical processing. These specimens are characterized by SEM (scanning electron microscopy; JOEL, Yokohama, Japan) with the image analysis to estimate the height (H), bottom diameter (B), and distancing space (D) of micro-corn unit cells in the acicular micro-textures. FT-IR (Fourier transform infra-red) spectroscopy (Shimazu; Kyoto, Japan) is utilized to measure the absorbance spectrum of near-infrared and far-infrared waves. In particular, the resonant peaks are quantitatively characterized to investigate the relationship between the unit size of acicular microtextures and the resonance peak position and intensity. Two types of heat radiation experiments are performed to describe the thermal transients on the objective plate by heat radiation from the micro-textured specimen, and to measure the reduction in the emitter temperature (T_E_) from the copper sheet temperature (T_Cu_). This reduction ratio (η = (T_Cu_ − T_E_)/T_Cu_) measures the heat radiation capacity at T_E_.

## 2. Materials and Methods

The acicular microtextures were deposited onto the oxygen-free copper sheet by electrochemical processing to prepare for the test devices with the controlled micro-cone textures. FT-IR was used to analyze the IR transmittance from the micro-textured test devices with reference to a bare copper substrate. Two types of heat radiation experimental setup were utilized to describe the heat radiation process. The heat radiation from the heated device to an objective plate was described by measuring the thermal transients on the top surface of the plate. The temperature difference between the copper plate and the microtextured layer surface was measured to discuss the heat transfer through the acicular microtextures.

### 2.1. Electrochemical Processing for Acicular Micro-Texturing

**Figure 2:**
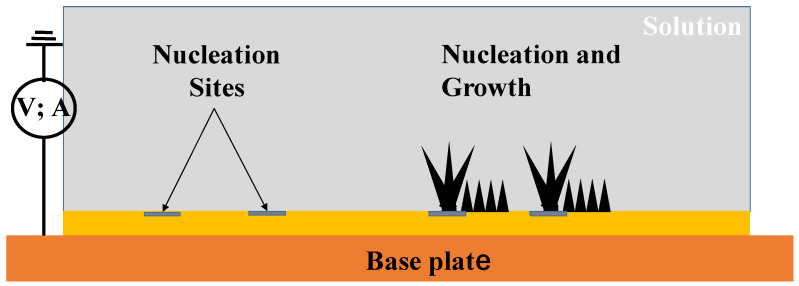
A schematic view on the electrochemical procedure with the use of electric field.

An oxygen-free copper (OFC; C1020) sheet specimen with the size of 50 mm × 50 mm × 0.5 mm was prepared for the electrochemical processing after the study [18,19]. Its surface roughness was measured to be 0.18 μm on average. Its mechanical properties were as follows: a Young’s modulus of 130 GPa, a yield stress of 290 MPa, and an ultimate strength of 310 MPa, which are comparable to the reported data in [20]. Figure 2 depicts the schematic view on the electrochemical procedure to synthesize and deposit the acicular micro-textures on the sheet. The nucleation sites were formed onto the copper sheet to selectively deposit the micro-corn units in a semi-regular alignment.

In the following experiments, the iron and nickel ionized solution was utilized to investigate the controllability of the unit cell size. Other ionized solutions than the Fe–Ni system are available in the present processing to control the unit cell size and topology.

### 2.2. FT-IR Spectroscopy

**Figure 3:**
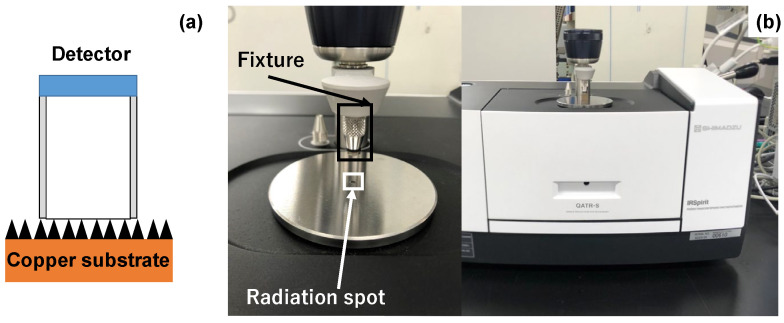
FT-IR measurement. (**a**) Schematic view on the FT-IR measurement and (**b**) overview on the measurement system for spectroscopic analysis.

The FT-IR measurement setup is schematically depicted in Figure 3a. The detector was placed onto the specimen to measure the transmittance of electromagnetic waves in the frequency range from the visible light to far-IR. As shown in Figure 3b, the FT-IR system (IR Spirit; Shimazu Co., Ltd., Kyoto, Japan) with the detector (QATR-S) was utilized for a spectroscopic measurement. The software (LabSolution IR) was also used to edit the measured raw data and to represent them as a transmittance spectrum as the function of the wave number (Λ).

### 2.3. Heat Radiation Experiment from Device to Objective Plate

**Figure 4:**
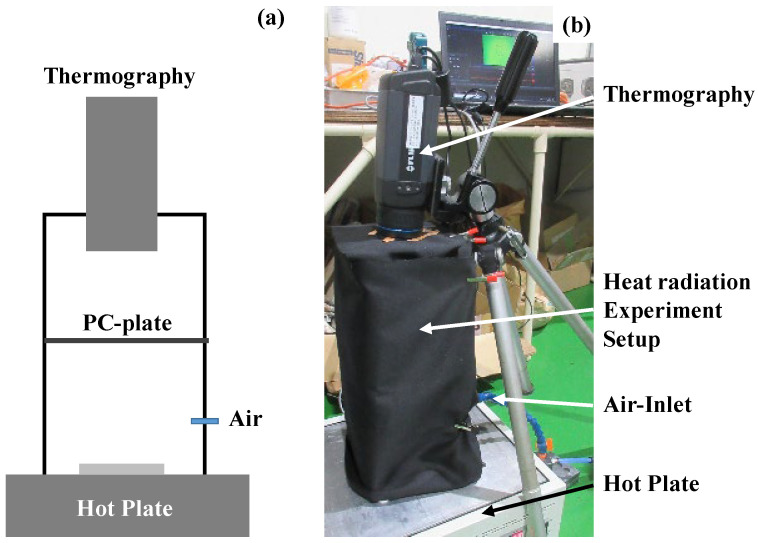
Heat radiation experimental setup. (**a**) Schematic view on the heat radiation from the specimen to the sample plate, in situ measured by the thermography and (**b**) overview on the experimental setup.

The heat radiation process advances from a high-temperature body to an objective one even through the vacuum. In this experiment, the test device was placed onto the hot plate with the holding temperature at 373 K. The black-colored polycarbonate (BC-PC) plate with a thickness of 2 mm was used as an objective body. As depicted in Figure 4a, thermography (FLIR-100; FLIR Co., Ltd. Frankfurt, Germany) was utilized to measure the thermal transients on the top surface of the BC-PC plate. This thermography was focused on the top surface only to measure the thermal transients on it. The whole setup was fully covered by the black cloth to minimize the optical noise. The forced air was supplied along the inside of the black cloth to exchange warm air with cooled air and to minimize the effect of heat convection on the temperature distribution measured on the BC-PC plate. When using the bare copper plate instead of a device, no temperate increase was detected on the top surface of the BC-PC plate. This thermal transient measurement was free from a convection heat transfer in the air below the bottom surface of the BC-PC plate. Figure 4b shows the overview of the experiment setup for the measurement of the thermal transients on the top surface of the BC-PC plate.

### 2.4. Measurement Setup on the Surface Temperature of Device

When the bare copper plate is placed onto the hot plate, no difference is detected between the holding temperature on the hot plate and the surface temperature on the copper plate. Under the heat radiation from the device, the surface temperature of the device is lower than the holding temperature on the hot plate due to the emittance of electromagnetic waves in IR wavelengths.

**Figure 5:**
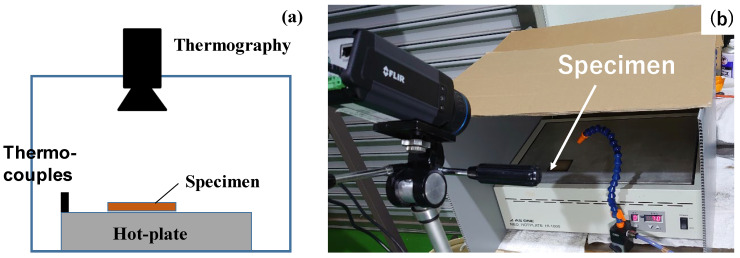
Experimental setup for measurement of the emitter temperature on the acicular micro-textured copper plate. (**a**) Schematic view on the setup and (**b**) overview of the setup.

The temperature distribution on the hot plate surface, including the specimens, is measured by the thermography, as illustrated in Figure 5a. The hot plate surface temperature (T_H_) was monitored by the thermocouple attached on the surface. The copper substrate temperature (T_Cu_) was also measured to certify that T_Cu_ ~ T_H_ during the experiment, as shown in Figure 5b. Thermography was also used and focused onto the device’s surface to measure its temperature (T_E_).

## 3. Results

The acicular microtextured device in a semi-regular alignment is characterized by scanning electron microscopy (SEM) to estimate the average unit sizes of the acicular micro-cones. The transmittance spectrum from near-IR to far-IR is measured to investigate the relationship between the resonance peaks and the average unit sizes of the acicular microtextures. Two types of heat radiation experiments are performed to demonstrate that the acicular microtextured sheet works as a heat transportation device.

### 3.1. Microstructure of Acicular Microtextures

**Figure 6:**
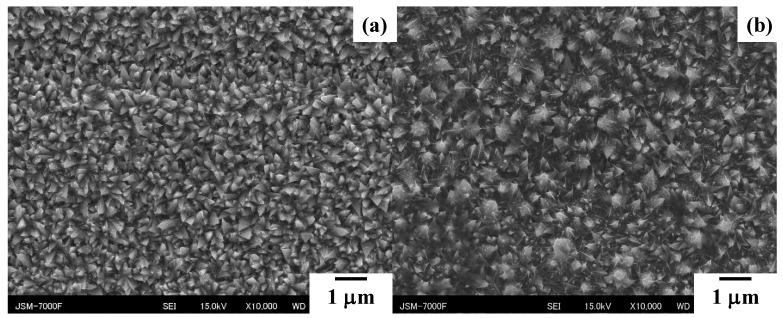
Comparison of SEM images on the growing acicular microtextures with the duration during the wet-plating process. (**a**) τ = 210 s and (**b**) τ = 630 s.

Under the deposition process during the electrochemical processing, each unit cell of acicular Fe-Ni crystal nucleates and grows on the substrate’s surface. This time, the evolution of the microtextures was described by comparing the SEM images of the microtextures at the duration (τ) of 210 s and 630 s, respectively. As compared in Figure 6, the size and height of the root are completely determined by the duration time. In Figure 6a, when τ is 210 s, B ranges from 0.1 to 0.4 μm and H is 0.5 μm on average. This tiny unit cell significantly grows, as compared between Figure 6a,b. When τ = 730 s, B ranges from 0.2 to 0.7 μm and H ~ 1 μm in Figure 6b.

**Figure 7:**
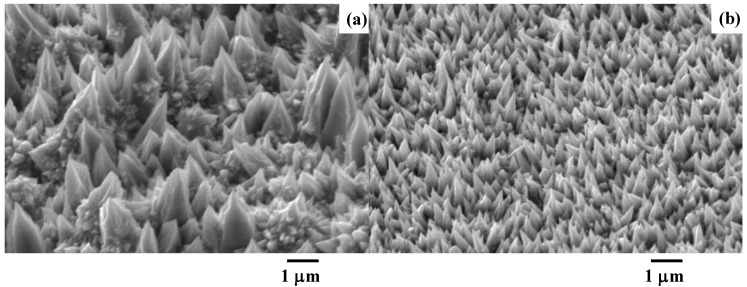
SEM image on the two types of acicular microtextures on the copper plate formed by the wet-plating. (**a**) Coarse acicular microtextures (LL-specimen) and (**b**) fine acicular microtextures (SS-specimen).

In this time evolution, since each micro-cone unit grows with self-similarity, the micro-cone size parameters {H, B, D} cannot be widely controlled. As depicted in Figure 7, both the total current and the current density are controlled to vary these micro-cone size parameters. In Figure 7a, H becomes higher than 3 μm, B becomes broader than 2 μm, and D widens due to the distribution of the micro-cone assemblies; this micro-textured device was called an LL-specimen. On the other hand, H, B, and D turn to be less than 1 μm in the SS-specimen, as depicted in Figure 7b. The small-sized micro-cones densely align by themselves on the substrate sheet. In the following experiments, the LL-specimen in Figure 7a is utilized to describe the acicular microtexturing effect on the heat radiation process.

Although atomic force microscopy (AFM; Shimazu, Kyoto, Japan) is effective for precisely measuring the three-dimensional profile of each micro-cone, SEM analysis in the above is suitable for a statistical evaluation on the micro-cone sizes.

### 3.2. Geometric Characterization on the LL-Specimen

**Figure 8:**
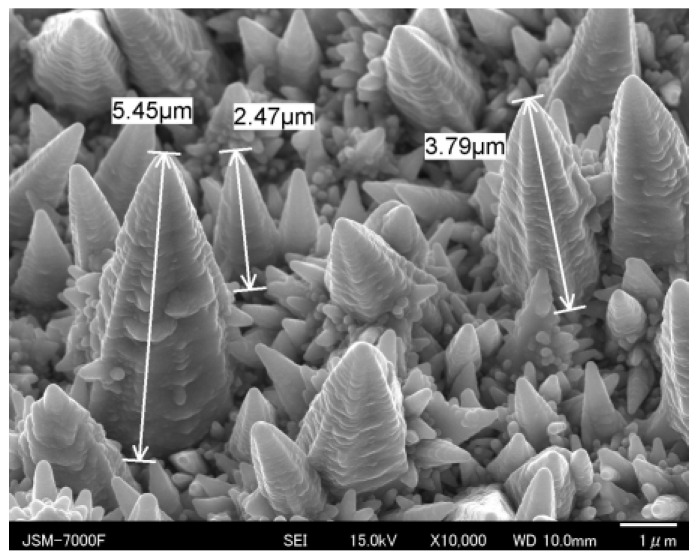
Local distribution of micro-cones in the LL-specimen, measured by SEM.

SEM was also utilized to characterize the semi-regular alignment of micro-cone units and to measure the statistical distributions of {H, B, D} by image processing. Figure 8 shows a three-dimensional SEM image on the micro-cone units of the LL-specimen in a local distribution with a skew angle of 30°. The micro-cone units with various heights distribute in a semi-regular way on the surface of the acicular microtextured device. Through this image analysis, the statistical distributions of {H, B, D} were obtained to characterize the semi-regular micro-cones in the acicular micro-texturing.

**Figure 9:**
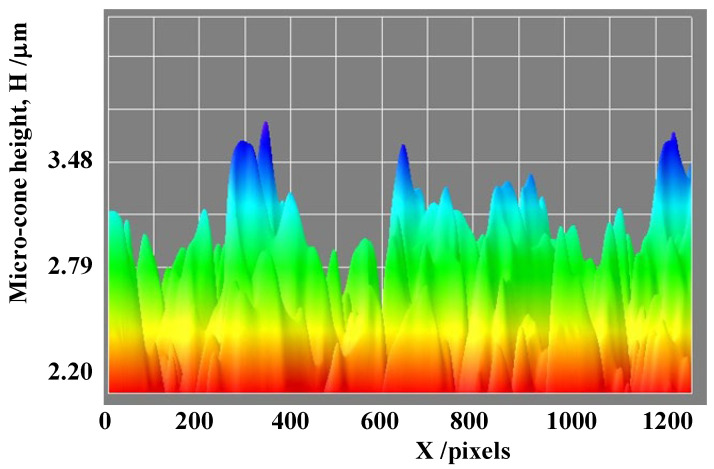
Micro-cone unit height distribution along the scanning line, X, for the LL-specimen.

Among the characteristic profiles of {H, B, D} for micro-cone units, the micro-cone height profile along the scanning line was depicted in Figure 9. In the image processing, each micro-cone height was digitized in each pixel range along the scanning direction. In reference to the height measurement in Figure 8, these digitized data were transformed to the height data. The originally measured profile consists of the bimodal distributions with an average height of H_ave_ = 3.3 μm in the blue-colored range and H_ave_ = 2.7 μm in the green-colored range, respectively, in Figure 9.

### 3.3. Transmittance Spectrum by FT-IR Analysis

**Figure 10:**
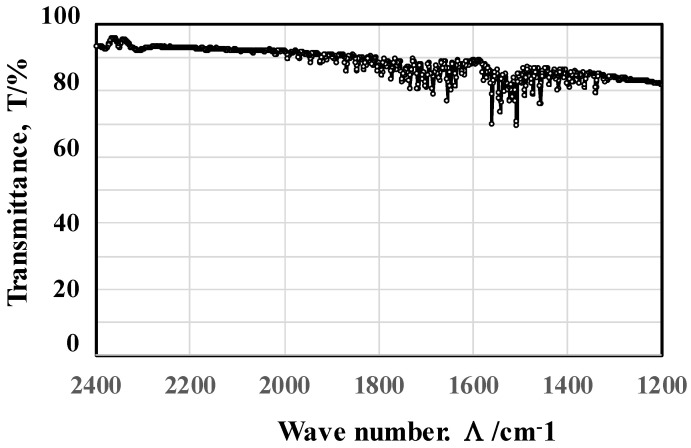
The transmittance spectrum measured by FT-IR in the range of wave number from 2400 cm^−1^ to 1200 cm^−1^ or in the range of wavelength from 4.2 μm to 8.4 μm for the LL-specimen.

FT-IR measurement and analysis was performed to describe the transmittance spectrum of the LL-specimen in the range of the wave number (Λ) from 2400 cm^−1^ to 1200 cm^−1^, or in the rage of the wavelength (λ) from 4.2 μm to 8.4 μm. Despite the fact that the baseline of the measured spectrum gradually decreases with a decreasing Λ or increasing λ, two absorbance bands were noticed at Λ_1_ = 1700 cm^−1^ with its deviation of ΔΛ_1_ = ±100 cm^−1^, and at Λ_2_ = 1500 cm^−1^ with its deviation of ΔΛ_2_ = ±100 cm^−1^, as shown in Figure 10.

Five positions were selected for FT-IR analysis to analyze the difference in the measured spectra among the FT-IR measurement positions on the same microtextured device.

**Figure 11:**
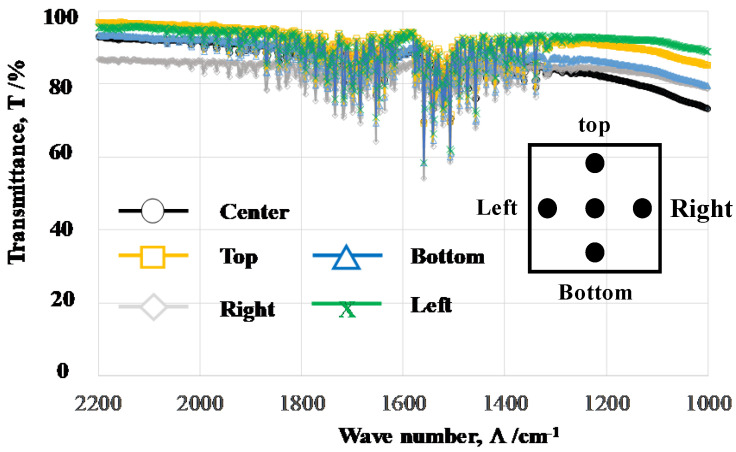
Measurement of the transmittance diagrams for LL-specimen at different five positions on the acicular microtextured surface of LL-specimen.

Figure 11 compares the measured spectrum at each position on the same device. Two absorbance bands around Λ_1_ and Λ_2_ were commonly detected in all measurements, irrespective of the positions. This implies that the FT-IR diagram in Figure 10 is intrinsic to the acicular microtextures in Figure 7a, Figure 8 and Figure 9. The relationship between the geometric configuration in acicular microtextures and the absorbance band widths from near-IR to far-IR is discussed later.

### 3.4. Heat Radition from Acicular Textured Device to Black-Colored PC Plates

The LL-specimen was placed onto the hot plate in the experimental setup, as shown in Figure 4. Thermography was focused at the center of the top surface of the BC-PC plate. The hot plate was switched on to retain its surface temperature by 373 K. Under forced air cooling to minimize the influence of the heat convention process on the measurement, the temperature transients on the top surface of the BC-PC plate were measured by thermography. Using the bare copper plate as a reference, no temperature rise, or ΔT = 0, was detected on the top of the BC-PC plates.

**Figure 12:**
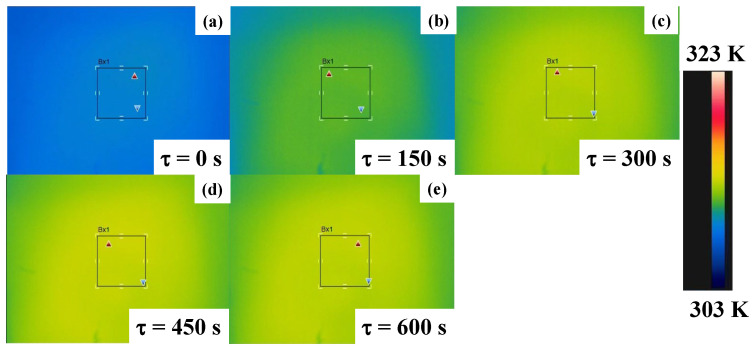
Time evolution of the temperature distribution on the top surface of BC-PC plate by the measurement through the thermography. (**a**) Initial temperature, (**b**) τ = 150 s, (**c**) τ = 300 s, (**d**) τ = 450 s, and (**e**) τ = 600 s.

Figure 12 depicts the time evolution of the temperature distributions measured on the top surface of the BC-PC plate. In the early stage of τ < 150 s, the center of the top surface was first heated and the temperature spread to either ends of the plate with time. This temperature rise homogenized all over the top surface with the duration in the radiation. At τ = 600 s, most of the spots on the top surface were homogeneously heated by ΔT ~ 4 K.

If the top plate was heated via the convection heat transfer by the warmed air below the BC-PC plate, the temperature distribution could be much more homogeneous even in the early stage of thermal transients. The time evolution of the temperature distribution in Figure 10 reveals that the heat convection by air blow below the BC-PC plate has no influence on the measured temperature distributions.

**Figure 13:**
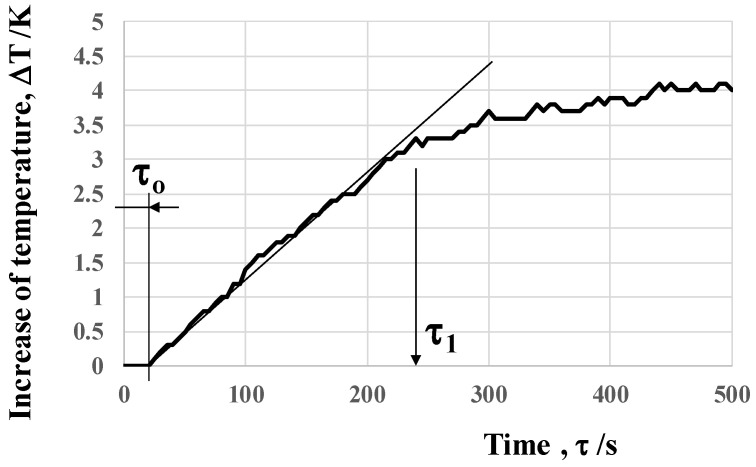
Variation in the maximum temperature at the focused center point in BC-PC with time by the heat radiation process.

The time evolution of the maximum temperature rise on the top surface of the BC-PC plate was monitored to describe the thermal transient response in this heat radiation. As depicted in Figure 13, the temperature starts to rise on the top surface after the incubation time (τ_0_), e.g., τ_0_ = 20 s. After this incubation, the temperature commences to increase linearly with time for τ_0_ < τ < τ_1_ (= 240 s). After τ_1_, the maximum temperature rise gradually saturates with time to be 4 K at τ = 500 s.

### 3.5. Reduction in Surface Temeprature on the Microtextured Device

**Figure 14:**
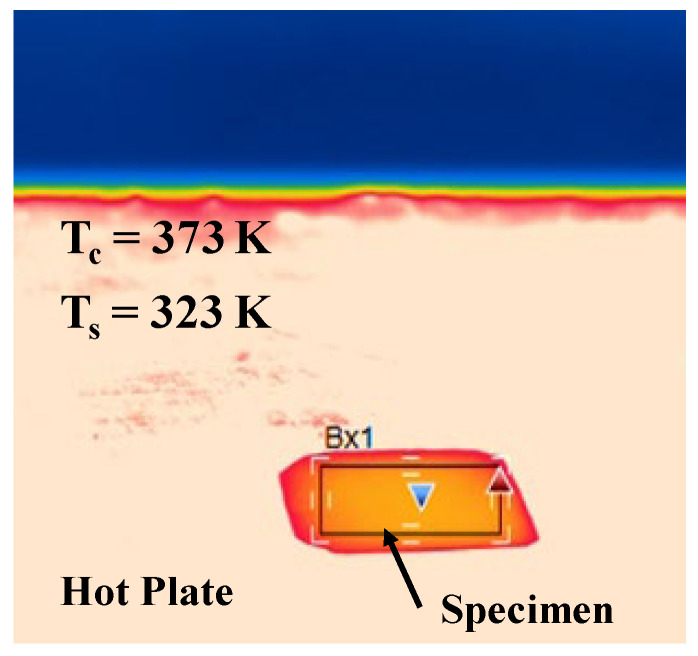
Comparison of the surface temperature between the bare copper and acicular micro-textured specimens, placed on the hot plate at 373 K.

The experimental setup in Figure 5 was utilized to describe the reduction in the surface temperature at the acicular microtextured emitter. The thermography was focused onto the device surface. Figure 14 shows the thermal distribution on the hot plate, on which the LL-specimen was placed. The hot plate temperature was held constant by 373 K (=100 °C). The surface temperature was measured to be 323 K; it was lower than the heat source temperature by 50 K.

This reduction in the device surface temperature (T_E_) from the hot plate’s temperature (T_H_) or the copper substrate’s temperature (T_Cu_) was obtained from the heat flux balance among the input, the output, and the heating of device. The input heat flux (q_input_) from the hot plate to the device is determined by the heat conduction through the copper substrate. The output heat flux (q_output_) is also governed by the heat radiation from the acicular microtextured layer and by the heat convection transfer through this layer. Then, T_E_ reduces from T_Cu_ by the heat balance against (q_input_ − q_output_). In the experimental setup in Figure 14, T_E_ = 50 °C and T_Cu_ = 100 °C and the ratio of η = (T_Cu_ − T_E_)/T_Cu_ reaches 50%. This implies that heat radiation has a significant contribution in heat flux through the acicular microtextured device.

## 4. Discussion

The FT-IR measurement works as a reliable tool to describe the absorbance of electromagnetic waves from near-IR to far-IR by a textured device and characterizes the unit cell size effect on the heat radiation mechanism. We assume that the resonance of electromagnetic waves occurs at a wavelength of λ_1_ and λ_2_ in the presence of microtextures with a height of H_1_ = λ_1_/2 and H_2_ = λ_2_/2 after the studies in [13,14]. A shown in Figure 10 and Figure 11, the two deepest valleys in the transmittance were seen at λ_1_ = 10^4^/Λ_1_ = 5.9 μm and λ_2_ = 10^4^/Λ_2_ = 6.7 μm for Λ_1_ = 1700 cm^−1^ and Λ_2_ = 1500 cm^−1^, respectively. In Figure 8 and Figure 9, the acicular microtextures have two groups of micro-cone units with H_ave_ = 2.7 μm and 3.3 μm, respectively. Since H_1_ = 2.7 μm ~ λ_1_/2 (=2.9 μm) and H_2_ = 3.3 μm ~ λ_2_/2 (=3.4 μm), the measured IR absorbance or IR emission are induced by the resonance of electromagnetic waves among the heated micro-cones with an average height of H_1_ and H_2_.

Let us consider the correspondence of the deviation of the micro-corn heights (H_dev_) to the width of the absorbance bands. In Figure 9, H_dev_ is estimated to be ±0.25 μm; then, the deviation of the resonant wavelength is calculated to be Δλ_dev_ = ±0.5 μm. From this Δλ_dev_, ΔΛ_dev_ is estimated to be ±100 cm^−1^. This estimate is just equivalent to the measured deviations (ΔΛ_1_ and ΔΛ_2_ = ±100 cm^−1^) of the absorbance band widths in Figure 10 and Figure 11. This implies that the band width of the measured absorbance spectra in Figure 10 and Figure 11 is characteristic of the micro-cone height deviation under the semi-regular alignment of the acicular microtextures in Figure 8 and Figure 9. The other two geometric parameters of B and D in the micro-cone unit contribute to the resonance of electromagnetic waves. Through the control of regularity in the micro-cone unit geometry and its alignment, the effect of H, B, and D in the microtextures on the near-IR and far-IR resonance in the absorbance and emission is quantitatively analyzed by the present method.

The absorbed electromagnetic waves from near-IR and far-IR by the acicular microtextures are emitted to the outside for heat radiation. In case of the experimental setup in Figure 4, these radiated waves are wasted in partially heating the air, but some waves reach the bottom surface of the BC-PC plate. Through the heat conduction in the PC plate from its bottom to top surfaces, the measured temperature starts to increase on the top surface. The incubation duration (τ_0_) in Figure 11 corresponds to the delay by this heat conduction in PC. The measured temperature on the top surface of the BC-PC plate gradually spreads from its center to every end through the heat conduction in the PC plate as shown in Figure 10. The linear increase in the maximum temperature at the center with the time depicted in Figure 11 explains that the radiated heat is transported from the back to the top surfaces of the BC-PC plate with a constant heat conductance.

This linearly increasing temperature at the top surface during the early stage gradually saturates in time. This saturation implies that three heat-transfer processes approach a stationary state in the experimental system in Figure 4. The heat flux by radiation, the heat loss in the air, and the heat conduction at the PC plate are balanced at the saturated temperature distribution on the top surface of the PC plate. When the hot plate temperature is held at 100 °C, the BC-PC plate is heated from its initial temperature of 30 °C to 34 °C in this experimental setup.

Through heat radiation from the acicular microtextures, their surface temperature (T_E_) decreases from the high-temperature heat source (T_Cu_). In the experimental setup in Figure 12, T_cu_ was held constant by 100 °C (or 373 K) on the hot plate, but T_E_ significantly reduced to 50 °C (or 323 K). After [14], the ratio (r) of the radiation emitter temperature decreased to the high-temperature source; it was only r = 10%. This high r up to 50% implies that acicular microtexturing is more suitable to a heat radiation device than the micro-cavity textures in [14].

Consider that the thickness (h) of the acicular microtextures, including the micro-cone unit height, is only 5 μm. Neglecting the heat conduction of the copper plate for simplicity, the temperature gradient across the microtextures is calculated by (T_Cu_ − T_E_)/h. This reaches up to 1 × 10^7^ K/m. This huge temperature gradient drives the emission of IR electromagnetic waves from the acicular microtextured surface with the wavelength of 2 × (H_ave_ ± H_dev_).

The present acicular microtextures are made from an iron and nickel alloy with the aid of electro-chemical processing. As stated in [19], other alloying systems are available for each application. In addition, the meta-material design is also available in acicular microtexturing by using the surface treatment and a thin-film coating.

## 5. Conclusions

The acicular micro-cone-textured sheet with a semi-regular alignment was proposed as a heat transportation device in a low-temperature condition. Its geometric configuration was characterized by SEM to describe the statistical profile of its unit cell size and pitch with their average and deviation. In particular, the unit cell height distributes by the bimodal profile in this semi-regular alignment of micro-corn cells. The LL-type specimen was fabricated to obtain a bimodal height profile with two average heights of H_1_ = 2.7 μm and H_2_ = 3.3 μm, respectively. The transmittance spectrum by FT-IR proves that near-IR and far-IR electromagnetic waves are emitted with selective wavelengths of λ_1_ ~ 2 × H_1_ and λ_2_ ~ 2 × H_2_, respectively. The deviation in the micro-cone height around each average height of H_1_ and H_2_ induces the absorbance band width in the transmittance spectrum.

The heat radiation experiment demonstrates that the top surface of the objective polycarbonate plate is heated by heat radiation from the acicular micro-textured specimen to its bottom surface and by heat conduction through the polycarbonate plate’s thickness. This low-temperature heat radiation plays the role of heat transportation from the high-temperature source to the objective media. The difficulty of a heat transfer from fuel cell-engine radiators is solved by this low-temperature heat radiation device.
